# Bloody Diarrhea in a 27-year-old Man with Adult-onset Still’s Disease

**DOI:** 10.31662/jmaj.2023-0103

**Published:** 2023-11-16

**Authors:** Ken Nagahata, Kazuyuki Murase, Masatoshi Kanda, Hiroki Takahashi

**Affiliations:** 1 Department of Rheumatology and Clinical Immunology, Sapporo Medical University School of Medicine, Sapporo, Japan; 2 Department of Medical Oncology, Sapporo Medical University School of Medicine, Sapporo, Japan

**Keywords:** adult-onset Still’s disease, bloody diarrhea, ulcerative colitis

## Abstract

A 27-year-old man presented with quotidian fever, rash, knee arthralgia, sore throat, and bloody diarrhea. Laboratory findings showed neutrophilia, elevated CRP, ferritin, and liver enzyme levels, and decreased hemoglobin levels. Radiological investigations revealed splenomegaly, systemic lymphadenopathy, thickening of the descending colon wall, and an abnormal uptake in the bone marrow and spleen as seen in F-fluorodeoxyglucose positron emission tomography. Malignant lymphoma was initially suspected, but biopsies showed no malignant findings. Colonoscopy revealed mucosal friability, erosions, and shallow ulcers, and pathological findings included crypt abscesses suggestive of either acute infectious colitis or inflammatory bowel disease. The patient was eventually diagnosed with adult-onset Still’s disease (AOSD) and started on prednisolone, which resolved bloody diarrhea, leading to the diagnosis of comorbid ulcerative colitis (UC). The combination of AOSD and UC presents a diagnostic challenge due to overlapping symptoms. An accurate diagnosis requires careful exclusion of other diseases and a comprehensive assessment.

## Introduction

Adult-onset Still’s disease (AOSD) is a rare autoinflammatory disorder of unknown origin characterized by quotidian fevers, arthritis, and an evanescent rash, affecting various organs. Infection, rheumatic diseases, malignancy, and drug reactions must be carefully excluded before a definitive diagnosis. Gastrointestinal symptoms may occur in up to 20% of patients with AOSD ^(1)^.

Ulcerative colitis (UC) is an inflammatory bowel disease (IBD) characterized by relapsing and remitting episodes of colitis, usually accompanied by bloody diarrhea. The diagnosis of UC may be difficult as it can mimic bacterial or drug-induced colitis on endoscopy. UC sometimes presents as a fever of unknown origin. Extraintestinal manifestations of UC include primary sclerosing cholangitis, uveitis, and erythema nodosum.

The complications of both diseases have not been widely reported. We report the case of a 27-year-old patient diagnosed with AOSD comorbid with UC with diagnostic difficulties.

## Case Report

A 27-year-old man presented with quotidian fever (up to 39.5°C), an evanescent rash and persistent pruritic erythema on the extremities, knee arthralgia, a sore throat, and bloody diarrhea occurring 4-5 times a day for a month. His medical history was significant for no abdominal pain. He had taken naproxen intermittently for one month for fever and arthralgia. Physical examination revealed swollen and tender joints in his knees and pruritic erythema on the extremities, but no abdominal tenderness. Laboratory investigations showed leukocytosis with a predominance of neutrophils (28.2 × 10^3^/μL); and elevated CRP (16 mg/dL), serum ferritin (17808 μg/L), and transaminase levels (AST 92 U/L, ALT 158 U/L). In addition, his hemoglobin levels were decreased (11.8 g/dL), and his antinuclear antibody and rheumatoid factor, as well as his blood culture results, were negative.

**Figure 1.**
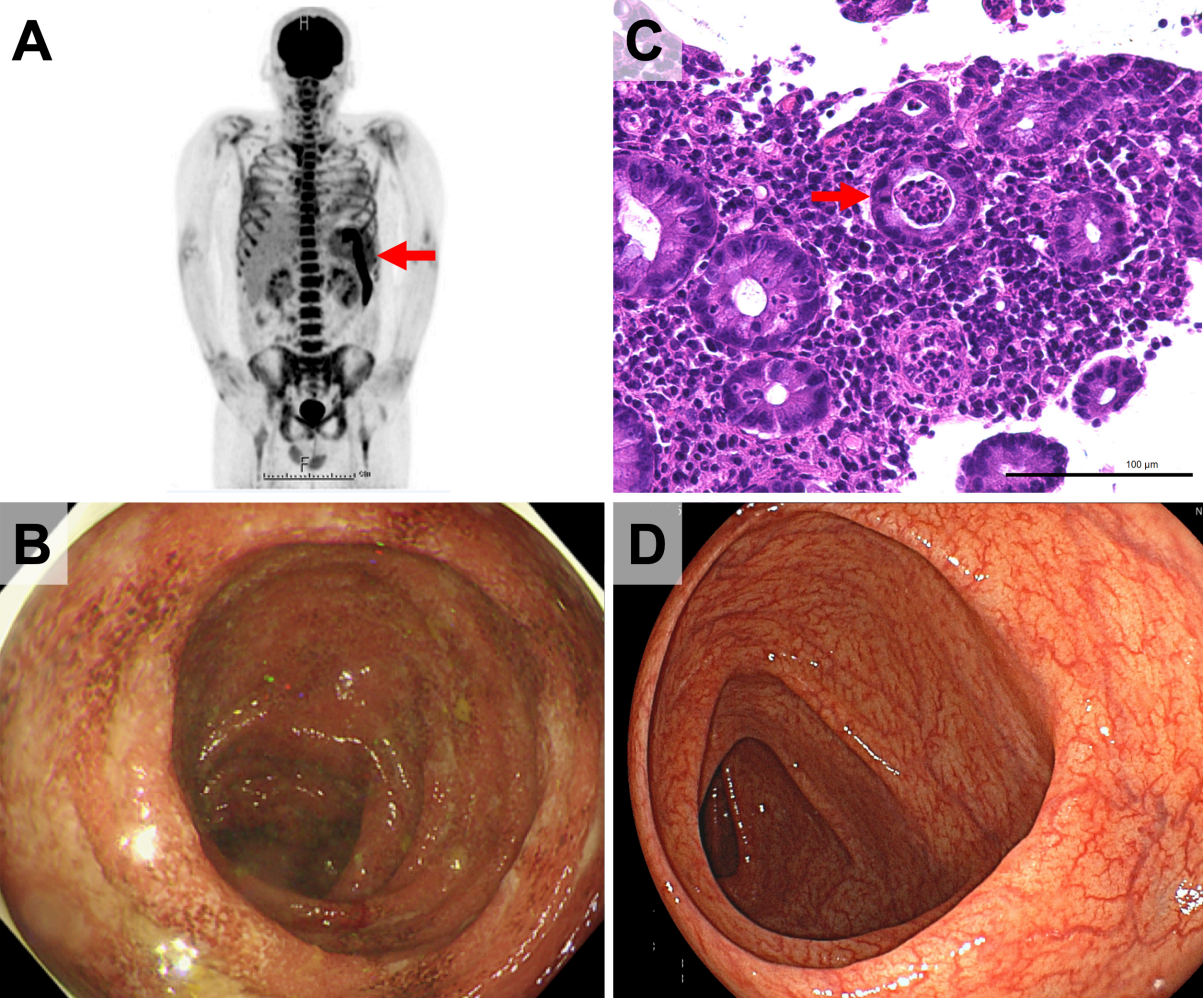
(A) ^18^F-fluorodeoxyglucose positron emission tomography and computed tomography revealing high uptake in the spleen, bone marrow, systemic lymph nodes, and splenic flexure of the colon (arrow). (B) Colonoscopic images showing mucosal friability, erosions, and multiple shallow ulcers in the area from the transverse colon to the rectum. (C) Histological evaluation revealing dense mucosal inflammatory infiltration and crypt abscesses (arrow). (D) Four-month follow-up colonoscopy showing the improvement of the colitis.

Contrast-enhanced computed tomography revealed splenomegaly, systemic lymphadenopathy, and descending colonic wall thickening, while ^18^F-fluorodeoxyglucose (FDG) positron emission tomography and computed tomography revealed high uptake in the spleen, bone marrow, systemic lymph nodes, joints, and the splenic flexure of the colon (Figure 1A). Initially, intestinal metastasis from malignant lymphoma was suspected. However, biopsies of the spleen, bone marrow, and skin showed no malignant findings. Histopathology of pruritic erythema revealed perivascular inflammatory cell infiltration and dyskeratotic cells in the upper layer of the epidermis.

A colonoscopy revealed mucosal friability, erosions, and shallow ulcers in multiple areas from the transverse colon to the rectum (Figure 1B). Histological evaluation revealed dense mucosal inflammatory infiltration and crypt abscesses (Figure 1C), suggesting either acute infectious colitis or IBD, particularly UC. Tissue culture results were negative.

First, AOSD was diagnosed based on the Yamaguchi criteria because there are no obvious findings of infection, malignancy, or other rheumatic diseases. Significantly high levels of ferritin, dyskeratotic cells in the epidermis, and abnormal FDG uptake in the bone marrow and spleen supported the diagnosis. Treatment was initiated with prednisolone (1 mg/kg/day), and the patient’s bloody diarrhea immediately resolved without antibiotic therapy, leading to the diagnosis of comorbid UC. At the 4-month follow-up colonoscopy, the colitis had improved (Figure 1D).

## Discussion

The combination of AOSD and IBD was a diagnostic dilemma. There have only been five case reports that describe AOSD complicated by IBD (Table 1) ^(2), (3), (4), (5), (6)^. Of these, AOSD preceded IBD chronologically in three cases and vice versa in two cases. It is particularly difficult to make an accurate diagnosis when they occur at the same time of onset, as in this case. A challenge in diagnosing combined AOSD and IBD is that the symptoms of one can mimic those of the other. For example, several symptoms, including fever, arthritis, or abdominal pain, can be described in both AOSD and IBD.

**Table 1.** Cases of Adult-Onset Still’s Disease Complicated by Inflammatory Bowel Disease.

Case	Age (years)	Gender	Order	Duration	Follow up	Year, (Reference)
Present case	27	M	AOSD/UC	-	18 months	-
1	31	M	CD→AOSD	9 years	Not mentioned	2003, ^(2)^
2	30	F	CD→AOSD	7 years	Not mentioned	2010, ^(3)^
3	38	M	AOSD→CD	14 years	Not mentioned	2013, ^(4)^
4	37	M	AOSD→UC	1 year	2 weeks	2017, ^(5)^
5	65	F	AOSD→CD	45 years	Not mentioned	2021, ^(6)^

AOSD: adult-onset Still’s disease, CD: Crohn’s disease, F: female, M: male, UC: ulcerative colitis.

However, transient erythema and sore throat have a high positive likelihood ratio (PLR) for AOSD (24.97 and 4.86, respectively). When combined with rash, sore throat, and leukocytosis, the PLR is higher (28.97) ^(7)^. In addition, hyperferritinemia (>1250 μg/L) combined with the Yamaguchi criteria had a high specificity (99.3%) for AOSD ^(8)^.

Abnormal FDG uptake in the bone marrow and spleen has been found in most cases of AOSD ^(9)^, and dyskeratosis of the superficial skin has been reported as a feature of an atypical rash in AOSD ^(10)^.

Therefore, the diagnosis of AOSD was made in this case. On the other hand, pathological findings in the colon could only be explained by UC, leading to the diagnosis of UC complicated with AOSD.

The diagnosis of IBD based on endoscopic evaluation and intestinal histopathology may occasionally be difficult, especially in patients with AOSD, due to the high risk of frequent exposure to infections and drugs, including non-steroidal anti-inflammatory drugs or antibiotics ^(8)^. Culture results and treatment response should also be considered for an accurate diagnosis.

Clinicians need to carefully exclude other diseases and perform a comprehensive assessment to make an accurate diagnosis for patients with rare multidisciplinary comorbidities.

## Article Information

### Conflicts of Interest

None

### Acknowledgement

We would like to thank Editage (www.editage.com) for the English-language editing.

### Author Contributions

KN worked on the manuscript and designed the figures and tables. KM aided in interpreting the results. MK and HT supervised the study. All authors substantially contributed to the revision of the manuscript drafts. All authors have approved the submitted version of the manuscript and agreed to be accountable for any part of the work.

### Informed Consent

Informed consent was obtained from the patient. An ethical review was not required for this single-case report.
